# The association between cumulative exposure to PM_2.5_ and DNA methylation measured using methyl-capture sequencing among COPD patients

**DOI:** 10.1186/s12931-024-02955-3

**Published:** 2024-09-09

**Authors:** Hyun Woo Ji, Jieun Kang, Hwan-Cheol Kim, Junghee Jung, Seon-Jin Lee, Ji Ye Jung, Sei Won Lee

**Affiliations:** 1 Division of Pulmonology, Department of Internal Medicine, National Health Insurance Service Ilsan Hospital, Goyang, Republic of Korea; 2 Division of Pulmonary and Critical Care Medicine, Department of Internal Medicine, Ilsan Paik Hospital, Inje University College of Medicine, Goyang, Republic of Korea; 3 Department of Occupational and Environmental Medicine, Inha University College of Medicine, Incheon, Republic of Korea; 4 Macrogen Inc., Seoul, Republic of Korea; 5 Environmental Disease Research Center, Korea Research Institute of Bioscience and Biotechnology, Daejeon, Republic of Korea; 6 Division of Pulmonary and Critical Care Medicine, Department of Internal Medicine, Severance Hospital, Yonsei University College of Medicine, Seoul, Republic of Korea; 7 Department of Pulmonary and Critical Care Medicine, Asan Medical Center, University of Ulsan College of Medicine, Seoul, Republic of Korea

**Keywords:** Chronic obstructive pulmonary disease, Particulate matter, DNA methylation, Respiratory health

## Abstract

**Background:**

Particulate matter with a diameter of < 2.5 μm (PM_2.5_) influences gene regulation via DNA methylation; however, its precise mechanism of action remains unclear. Thus, this study aimed to examine the connection between personal PM_2.5_ exposure and DNA methylation in CpG islands as well as explore the associated gene pathways.

**Methods:**

A total of 95 male patients with chronic obstructive pulmonary disease (COPD) were enrolled in this study. PM_2.5_ concentrations were measured for 12 months, with individual exposure recorded for 24 h every 3 months. Mean indoor and estimated individual PM_2.5_ exposure levels were calculated for short-term (7 days), mid-term (35 days), and long-term (90 days). DNA methylation analysis was performed on the blood samples, which, after PCR amplification and hybridization, were finally sequenced using an Illumina NovaSeq 6000 system. Correlation between PM_2.5_ exposure and CpG methylation sites was confirmed via a mixed-effects model. Functional enrichment analysis was performed on unique CpG methylation sites associated with PM_2.5_ exposure to identify the relevant biological functions or pathways.

**Results:**

The number of CpG sites showing differential methylation was 36, 381, and 182 for the short-, mid-, and long-term indoor models, respectively, and 3, 98, and 28 for the short-, mid-, and long-term estimated exposure models, respectively. The representative genes were *TMTC2* (*p* = 1.63 × 10^-3^, R^2^ = 0.656), *GLRX3* (*p* = 1.46 × 10^-3^, R^2^ = 0.623), *DCAF15* (*p* = 2.43 × 10^-4^, R^2^ = 0.623), *CNOT6L* (*p* = 1.46 × 10^-4^, R^2^ = 0.609), *BSN* (*p* = 2.21 × 10^-5^, R^2^ = 0.606), and *SENP6* (*p* = 1.59 × 10^-4^, R^2^ = 0.604). Functional enrichment analysis demonstrated that the related genes were mostly associated with pathways related to synaptic transmission in neurodegenerative diseases and cancer.

**Conclusion:**

A significant association was observed between PM_2.5_ exposure and DNA methylation upon short-term exposure, and the extent of DNA methylation was the highest upon mid-term exposure. Additionally, various pathways related to neurodegenerative diseases and cancer were associated with patients with COPD.

**ClinicalTrials.gov identifier:**

NCT04878367.

**Supplementary Information:**

The online version contains supplementary material available at 10.1186/s12931-024-02955-3.

## Introduction

Ambient air pollution has significant adverse effects on human health. Inhalation of particulate matter with a diameter of < 2.5 μm (PM_2.5_) increases the risk of various diseases, including respiratory disease, cardiovascular disease, endocrine disorders, and neurodegenerative disease [1, 2]. According to the Global Burden of Disease Study 2019, PM_2.5_ pollution is the leading level-4 risk factor for disability-adjusted life years (DALYs) among environmental and occupational risks, contributing 118 million DALYs and 4.14 million deaths in 2019, ranking seventh and sixth among all risk factors for DALYs and death, respectively. Therefore, PM_2.5_ pollution is a primary public health concern worldwide [3].

Upon inhalation, particulate matter (PM) poses a primary health risk. Chronic obstructive pulmonary disease (COPD) is a major health issue associated with PM and the third leading cause of death worldwide [4, 5]. PM_2.5_ significantly impact COPD through various mechanisms including epigenetic modification, exacerbating symptoms and influencing disease progression. Epigenetic modifications provide an important link between the environment and alteration in gene expression. Epigenetic changes are genetic modifications that impact gene activity without changing the DNA sequence through DNA methylation, posttranslational histone modification, histone variation, chromatin remodelling, or noncoding RNA [6]. DNA methylation is a key epigenetic modification involving the covalent addition of a methyl group to a cytosine (C) residue, and promoter methylation is correlated with gene expression silencing [7, 8]. Upon exposure to PM_2.5_, significant methylation changes in various genes related to inflammation, immune response, cell motility, and cell growth as well as death have been observed in human bronchial epithelial cells [9]. Additionally, a significant relationship has been reported between PM_2.5_ exposure and DNA methylation in inflammatory and immune responses among patients with COPD [10, 11].

However, previous studies have considered only small populations or targeted methylation changes only in specific genes. These studies relied on fixed outdoor monitoring stations with limited spatial resolution to estimate PM_2.5_ exposure and did not investigate variable exposure durations. Moreover, the exact mechanism by which PM_2.5_ exposure affects the human body, especially the respiratory system, is not yet fully understood. Therefore, this study aimed to determine the relationship between personal PM_2.5_ exposure and DNA methylation in known CpG islands (CGIs) and explore the functional pathways related to the relevant genes.

## Methods

### Study population

DNA methylation profiling was conducted on patients selected from a multicenter trial that assessed PM_2.5_ exposure in patients with COPD (ClinicalTrials.gov identifier: NCT04878367) [5, 12]. Briefly, the study included patients aged 40–79 years with a forced expiratory volume in 1 s (FEV_1_) < 80% of the predicted value, FEV_1_ / forced vital capacity (FVC) < 0.7, and respiratory symptoms. Indoor and outdoor PM_2.5_ levels were measured for 1 year, and the patients were followed up every 3 months. Blood samples were collected during the last visit. Only male patients were included in our analysis owing to the small number of female patients.

### Environmental measurements

PM_2.5_ exposure was measured in two ways, as described in previous studies [5]. Both outdoor and indoor PM_2.5_ concentrations were continuously monitored using ‘internet-of-things’-based devices (CP-16-A5; Aircok, Seoul, Republic of Korea) installed inside and outside of all participants’ houses. Additionally, gravimetric and light-scattering methods were employed to obtain more accurate indoor PM measurements. A mini-volume air sampler (model: KMS-4100; KEMIK, Seongnam, Republic of Korea), MicroPEM™ (RTI International, Research Triangle Park, NC, USA), and dust spectrometer (11-D; GRIMM Aerosol Technik Ainring GmbH & Co. KG, Ainring, Germany) were installed at the houses of participants for 24 h every 3 months to ensure comprehensive data collection. Moreover, participants maintained a time–activity diary documenting their time spent indoors and outdoors and carried a portable PM_2.5_-measuring device (Airbeam2; HabitatMap, Brooklyn, NY, USA) for 24 h before each follow-up visit every 3 months. Based on these data, individual PM_2.5_ exposure levels were estimated for each participant. The detailed estimation methods used are described in the Supplementary Material. In this analysis, we used both indoor PM_2.5_ concentrations and estimated individual PM_2.5_ exposure level. The average PM_2.5_ concentration was categorized according to three different time periods before the last visit when the blood sample was obtained. The time periods are as follows: short-term (7 days), mid-term (35 days), and long-term (90 days).

### DNA methylation profiling

#### Sampling and library construction

DNA methylation analysis was performed using the blood samples obtained after 1 year of follow-up. The fragmented genomic DNA was repaired and SureSelect Methyl-Seq Methylated Adapters (Agilent, Santa Clara, CA, USA) were ligated to the fragments. The adapter-ligated product was then PCR amplified, following which the final purified product was quantified and qualified. Target capture for DNA library was prepared according to the standard SureSelect Methyl-Seq Target Enrichment protocol (Agilent). Upon hybridization of the capture baits, the SureSelect Human Methyl-Seq kit (Agilent) captured 84.4 Mb of the human genome. Hybrids were captured on streptavidin beads and the captured genomic DNA was eluted. Unmethylated C residues were modified via bisulfite conversion, using the EZ DNA Methylation Gold kit (Zymo Research). The final libraries were sequenced using an Illumina NovaSeq 6000 System.

### Methylation calling and data preprocessing

Figure S1 in the Supplementary Material shows the analytical methods and workflow. After sequencing, the raw sequence reads were trimmed and aligned to the *Homo sapiens* hg19 reference genome using BSMAP (version 2.90). After the mapped reads were sorted and indexed, the PCR duplicates were removed. The methylation ratio at each cytosine position within the target region was subsequently extracted from the mapping results. The coverage profiles were calculated as C counts/effective CT counts for each cytosine in CpG, CHH, and CHG. Each cytosine locus in CpG, CHH, and CHG was annotated in terms of the functional location of each gene (promoter regions, exons, and introns), transcript ID, gene ID, strand, or CGI.

For data preprocessing, we selected only CpG sites with at least 10 CT counts at each site to obtain a more reliable methylation ratio. The methylation ratio data were normalized using the median scaling normalization method to reduce technical bias and better comparisons between the data samples.

### Model selection

We randomly selected 500 CpG sites and checked all the assumptions of the linear regression model in each case as shown in Supplementary Material (linearity between the independent and dependent variables, independence of observations, homoscedasticity (constant variance of residuals), and normality of residuals). To test these assumptions, we used the studentized Breusch-Pagan test for homoscedasticity and the Shapiro-Wilk normality test for normality on a randomly selected 500 CpGs. The data exhibited heteroscedasticity and non-normal residuals. Additionally, our data includes repeated measurements of fine particulate concentrations (indoor concentration and estimated individual exposure) over 7, 35, and 90 days. To account for these repeated measures and individual-level variations such as asthma history, history of coronavirus disease 2019 (COVID-19) infection status, and smoking history, we considered a mixed-effects model. The initial model specified a variance function for the repeated measures and included fixed effects for PM values, age, BMI, asthma history, and FEV_1_ predicted %, COVID-19 infection as a random effect. We found that specifying the variance function by asthma history rather than repeated measures resulted in a lower Alaike information criterion value, indicating a better model fit. Therefore, in the final model excluded the repeated measures variable and included short, mid, and long-term PM concentrations as fixed effects, with the variance function defined by asthma status.

### Identification of DNA methylation associated with PM exposure

We used mixed-effects models with generalized least squares model (‘gls’ function in R package ‘nlme’) to evaluate the associations between PM exposure and DNA methylation. The fixed effects of the model were age, BMI, asthma history, and FEV_1_ predicted %. The variance covariates were repeated-measure days (7, 14, 21, 35, and 90 days) or asthma history. The history of the COVID-19 infection was considered a random effect. We set a threshold of Benjamini–Hochberg false discovery rate-adjusted *p* value < 0.05 for assessing the genome-wide statistical significance of the fixed effect of PM exposure.

### Enrichment analysis

To further explore the biological function, cellular component, and molecular function of unique CpG methylation sites related to PM exposure, we conducted a gene set enrichment test based on Gene Ontology (GO), using gProfiler [13]. Additionally, pathway analysis was performed using the Kyoto Encyclopedia of Genes and Genomes (KEGG) database [14]. Adjusted *p*-values reported in the gProfiler result were derived using a one-sided hypergeometric test and corrected using the Benjamini–Hochberg method. Adjusted *p*-values from the KEGG results were derived using a two-sided modified Fisher’s exact test and corrected using the Benjamini–Hochberg method. Results from each enrichment test were considered significant when the adjusted *p*-values were < 0.05.

## Results

Among the 102 patients who participated in the trial [5, 12], 95 male patients provided blood samples, which were subsequently analyzed in the current study. The baseline patient characteristics are presented in Table 1. The mean age was 68.2 years, and the mean BMI was 28.3 kg/m^2^. Current and former smokers comprised 18.9% and 77.9% of all patients, respectively. Their mean post-bronchodilator FEV_1_ was 56.8% of the predicted value. The mean indoor PM_2.5_ concentrations were 8.8, 13.3, and 15.8 µg/m^3^ for the short-, mid-, and long-term periods, respectively. The estimated individual PM_2.5_ exposure levels were 10.0, 13.7, and 16.8 µg/m^3^ in the short-, mid-, and long-term periods, respectively.

**Table 1:** Baseline characteristics of study participants

Baseline characteristics	All (*n* = 95)
Age, years	68.2 ± 6.4
Sex, male	95 (100.0)
Smoking (pack-years)	37.0 ± 16.6
Current smoker	18 (18.9)
Former smoker	74 (77.9)
Never smoker	3 (3.2)
Body-mass index (kg/m^2^)	23.8 ± 3.8
Underlying asthma	3 (3.2)
Education level	
Middle school	27 (28.4)
High school	37 (38.9)
College	24 (25.3)
Graduate school	7 (7.4)
Monthly income (US dollars)	
≥ 4,600	8 (8.4)
3,000–4,599	15 (15.8)
1,500–2,999	18 (18.9)
700–1,499	18 (18.9)
< 700	23 (24.2)
Exacerbation during the past year	
Moderate	23 (24.2)
Severe	11 (11.6)
All (moderate-severe)	34 (35.8)
Lung function	
Post-BD FEV_1_/FVC (%)	54.9 ± 13.0
Post-BD FEV_1_ (%pred.)	56.8 ± 14.6
Post-BD FVC (%pred.)	82.1 ± 13.0
DL_CO_ (%pred.)	60.9 ± 18.2
Inhaler treatment	
LABA + LAMA	49 (51.6)
ICS + LABA + LAMA	35 (36.8)
LABA or LAMA	6 (6.3)
ICS + LABA	5 (5.3)
SGRQ-C	
Total	36.9 ± 20.6
Symptom	44.8 ± 21.6
Activity	48.2 ± 24.5
Impact	27.4 ± 22.6
CAT score	15.1 ± 8.4
mMRC grade	2.4 ± 1.1
PM_2.5_ levels (µg/m^3^)	
Estimated individual: short-term	10.0 ± 3.9
Indoor: short-term	8.8 ± 4.0
Estimated individual: mid-term	13.7 ± 4.9
Indoor: mid-term	13.3 ± 6.5
Estimated individual: long-term	16.8 ± 5.0
Indoor: long-term	15.8 ± 6.9

Data are presented as number (%) or mean ± standard deviation, unless otherwise indicated.

Abbreviations: BD, bronchodilator; FEV_1_, forced expiratory volume in 1 s; FVC, forced vital capacity; %pred, percent of the predicted value; DL_CO_, diffusing capacity of the lungs for carbon monoxide; LABA, long-acting beta-2 agonist; LAMA, long-acting muscarinic antagonist; ICS, inhaled corticosteroid; SGRQ-C, St. George’s Respiratory Questionnaire for patients with COPD; CAT, chronic obstructive pulmonary disease assessment test; mMRC, modified Medical Research Council; PM_2.5_, particulate matter less than 2.5 μm in diameter

**Fig. 1:**
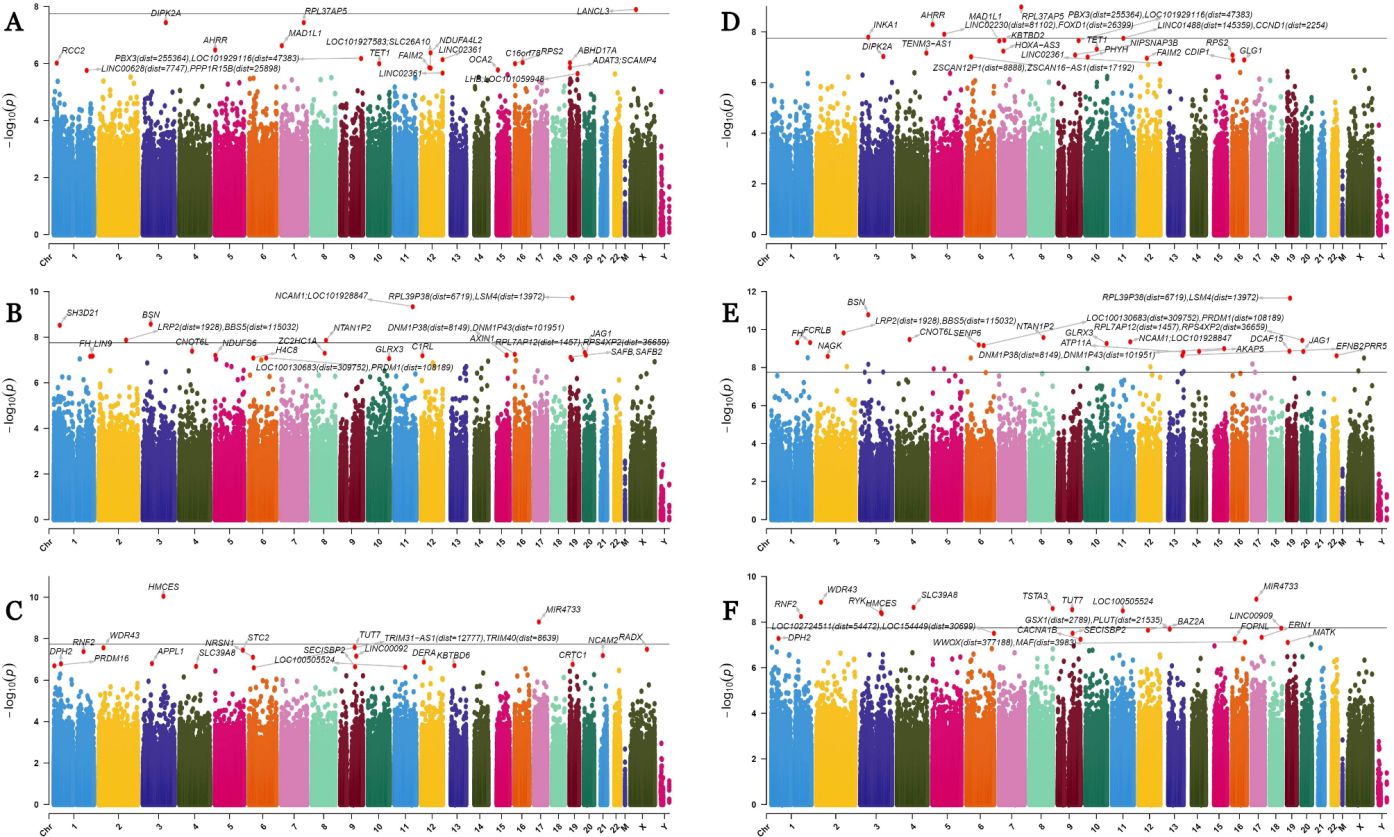
Manhattan plot indicating the associations between PM_2.5_exposure and DNA methylation. Every point corresponds to a CpG methylation site. The x-axis shows the chromosome of each CpG locus. The horizontal line corresponds to the Bonferroni-corrected threshold. (**A**) Short-, (**B**) mid-, and (**C**) long-term estimated individual exposure models. (**D**) Short-, (**E**) mid-, and (**F**) long-term indoor exposure models

The associations between DNA methylation and PM_2.5_ exposure were analyzed using a mixed-effects model, considering the PM_2.5_ measurement methods (indoor concentration and estimated individual exposure level) and periods (short-term, mid-term, and long-term). Age, BMI, asthma history, and FEV_1_ were considered as fixed effects and COVID-19 history as a random effect in each model. The number of CpG sites that showed significant differences in methylation upon PM_2.5_ exposure was 3, 98, and 28 in the short-, mid-, and long-term estimated individual exposure models, respectively, and 36, 381, and 182 in the short-, mid-, and long-term indoor models, respectively. The CpGs for each model are shown as a Manhattan plot in Fig. 1.

**Fig. 2:**
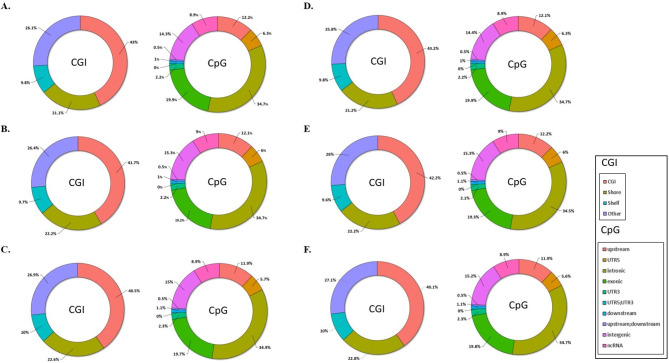
Positions of CpGs relative to CGIs and their functional genome distribution. (**A**) Short-, (**B**) mid-, and (**C**) long-term estimated individual exposure models. (**D**) Short-, (**E**) mid-, and (**F**) long-term indoor exposure models. CpG, 5′-C-phosphate-G-3′; CGI, CpG island

We characterized the positions of the differentially methylated CpGs relative to the CGI on the chromosome and determined their functional genomic distribution. Notably, 40.1–43.2% of the total CpGs were located within the respective CGI. The proportion of functional CpGs located within the promoter was 2 (66.7%), 36 (36.7%), and 6 (21.4%) in the short-, mid-, and long-term estimated individual exposure models, respectively, and 14 (38.9%), 132 (34.6%), and 46 (25.3%) in the short-, mid-, and long-term indoor models, respectively (Fig. 2).

The effects of PM exposure on CpG methylation were analyzed according to exposure duration (short-, mid-, and long-term). When the R-square of the model was limited to ≥ 0.5, 16 CpGs showed methylation differences (Table 2), all of which were significantly associated with mid-term PM_2.5_ exposure. Other differentially methylated CpGs (with an R-square between 0.4 and 0.5) are described in Table S1. Notably, the number of associations was the largest for mid-term exposure.

**Table 2:** Characteristics of differentially methylated CpG associated with PM_2.5_ exposure in the genome-wide methylation analysis

Chromosome	Locus	Gene	Region	CGI type	adj. *p* value	Marginal *R*^2^	Conditional *R*^2^
1q23.3	chr01-161696583	*FCRLB*	Exonic	CGI	1.46 × 10^− 4^	0.515	0.515
1q43	chr01-241682903	*FH*	Exonic	CGI	1.46 × 10^− 4^	0.516	0.516
2q31.1	chr02-170220972	*LRP2*, *BBS5*	Intergenic	Shore	1.35 × 10^− 4^	0.614	0.614
3p21.31	chr03-49708453	*BSN*	UTR3	Shelf	2.21 × 10^− 5^	0.606	0.606
4q21.1	chr04-78739828	*CNOT6L*	Intronic	CGI	1.46 × 10^− 4^	0.609	0.609
6q14.1	chr06-76311490	*SENP6*	UTR5	CGI	1.59 × 10^− 4^	0.604	0.604
8q11.21	chr08-48650995	*CEBPD*	Upstream	CGI	8.01 × 10^− 3^	0.501	0.501
10q26.3	chr10-131934635	*GLRX3*	Upstream	CGI	1.46 × 10^− 4^	0.623	0.623
12q21.1	chr12-72094870	*TMEM19*	UTR3	.	9.18 × 10^− 4^	0.515	0.515
12q21.31	chr12-83081152	*TMTC2*	UTR5	CGI	1.63 × 10^− 3^	0.656	0.656
13q34	chr13-115079970	*CHAMP1*	Upstream	CGI	1.37 × 10^− 3^	0.502	0.502
15q25.2	chr15-82824909	*DNM1P38*, *DNM1P43*	Intergenic	CGI	2.13 × 10^− 4^	0.511	0.511
19p13.12	chr19-14063293	*DCAF15*	UTR5	CGI	2.43 × 10^− 4^	0.623	0.623
19p13.11	chr19-18403074	*RPL39P38*, *LSM4*	Intergenic	CGI	6.10 × 10^− 6^	0.561	0.561
20p12.2	chr20-10654937	*JAG1*	Upstream	CGI	2.43 × 10^− 4^	0.583	0.583
20p13	chr20-4573410	*RPL7AP12*, *RPS4XP2*	Intergenic	CGI	1.46 × 10^− 4^	0.755	0.755

Abbreviations: CpG, 5′-C-phosphate-G-3′; PM_2.5_, particulate matter less than 2.5 μm in diameter; CGI, CpG island; UTR, untranslated region; ncRNA, non-coding ribonucleic acid

In particular, the R-square value was extremely high (> 0.6) in the promoter region that regulates the transcription of SUMO-specific peptidase 6 (*SENP6*, *p* = 1.59 × 10^− 4^, R^2^ = 0.604), glutaredoxin-3 (*GLRX3*, *p* = 1.46 × 10^− 4^, R^2^ = 0.623), transmembrane O-mannosyltransferase-targeting cadherins (*TMTC2*, *p* = 1.63 × 10^− 3^, R^2^ = 0.656), and DDB1 and CUL4-associated factor (*DCAF15*, *p* = 2.43 × 10^− 4^, R^2^ = 0.623) genes.

**Fig. 3:**
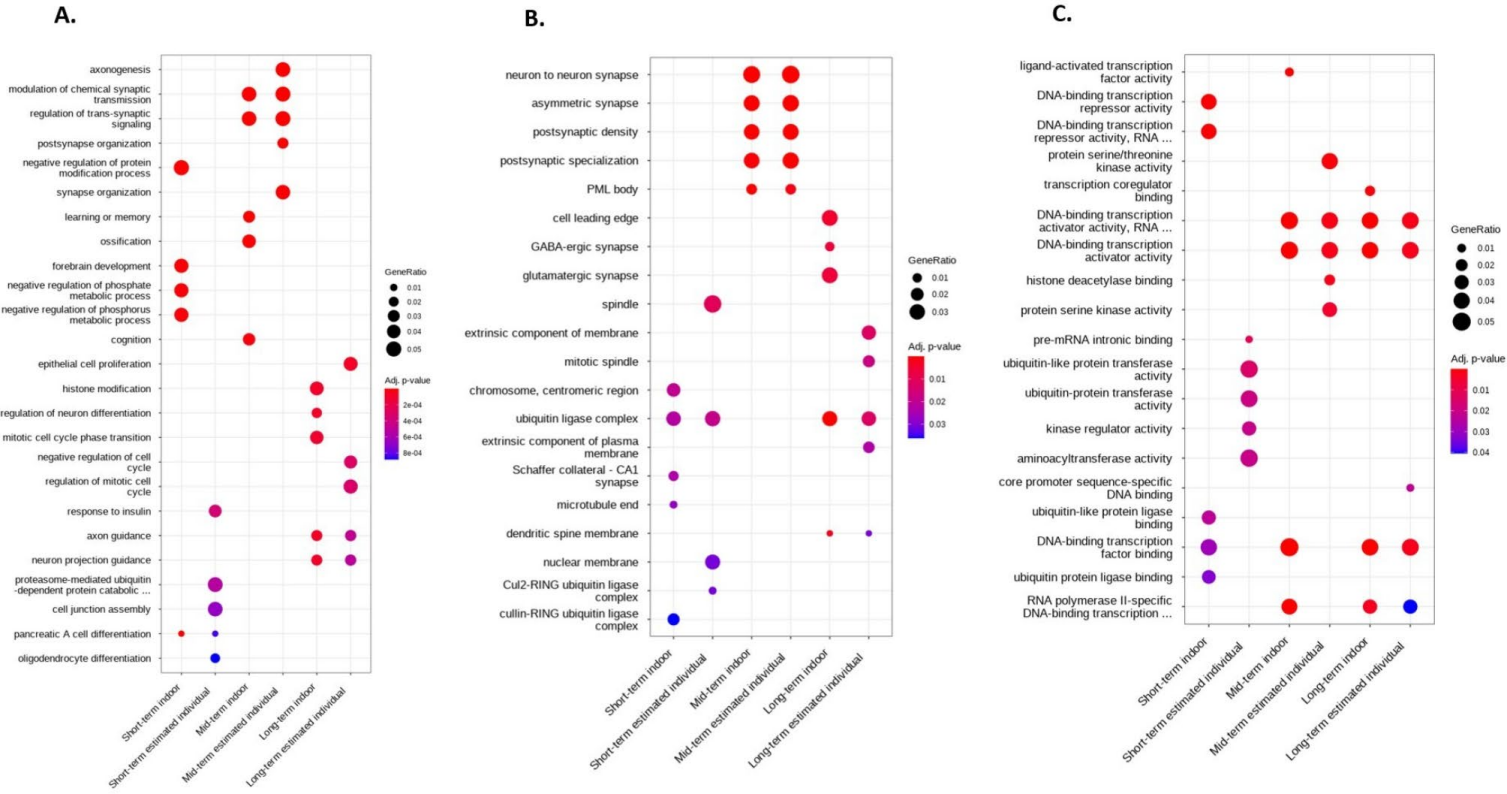
Results of Gene Ontology enrichment analysis. Top 5 Gene Ontology pathways displaying the most significant differences among the groups with different durations of estimated individual and indoor exposure to PM_2.5_. (**A**) Biological processes, (**B**) cellular components, and (**C**) molecular functions

GO-based gene set enrichment analysis was performed to further explore the biological processes, cellular components, and molecular functions related to genes that showed methylation differences depending on PM exposure duration (Fig. 3). This correlation varied depending on the PM_2.5_ exposure period. In the biological process category, genes related to trans-synaptic signaling, such as modulation of chemical synaptic transmission and regulation of trans-synaptic signaling, and axonogenesis, such as axon and neuron projection guidance, were enriched. In the cell component category, genes related to the ubiquitin ligase complex or synapses, such as neuron-to-neuron synapses, asymmetric synapses, postsynaptic density, and postsynaptic specialization were enriched. In the molecular function category, genes related to DNA-binding transcription activator activity and DNA-binding transcription factor binding were enriched. The enrichment analysis identified a total of 244 GO terms (Figure S2).

**Fig. 4:**
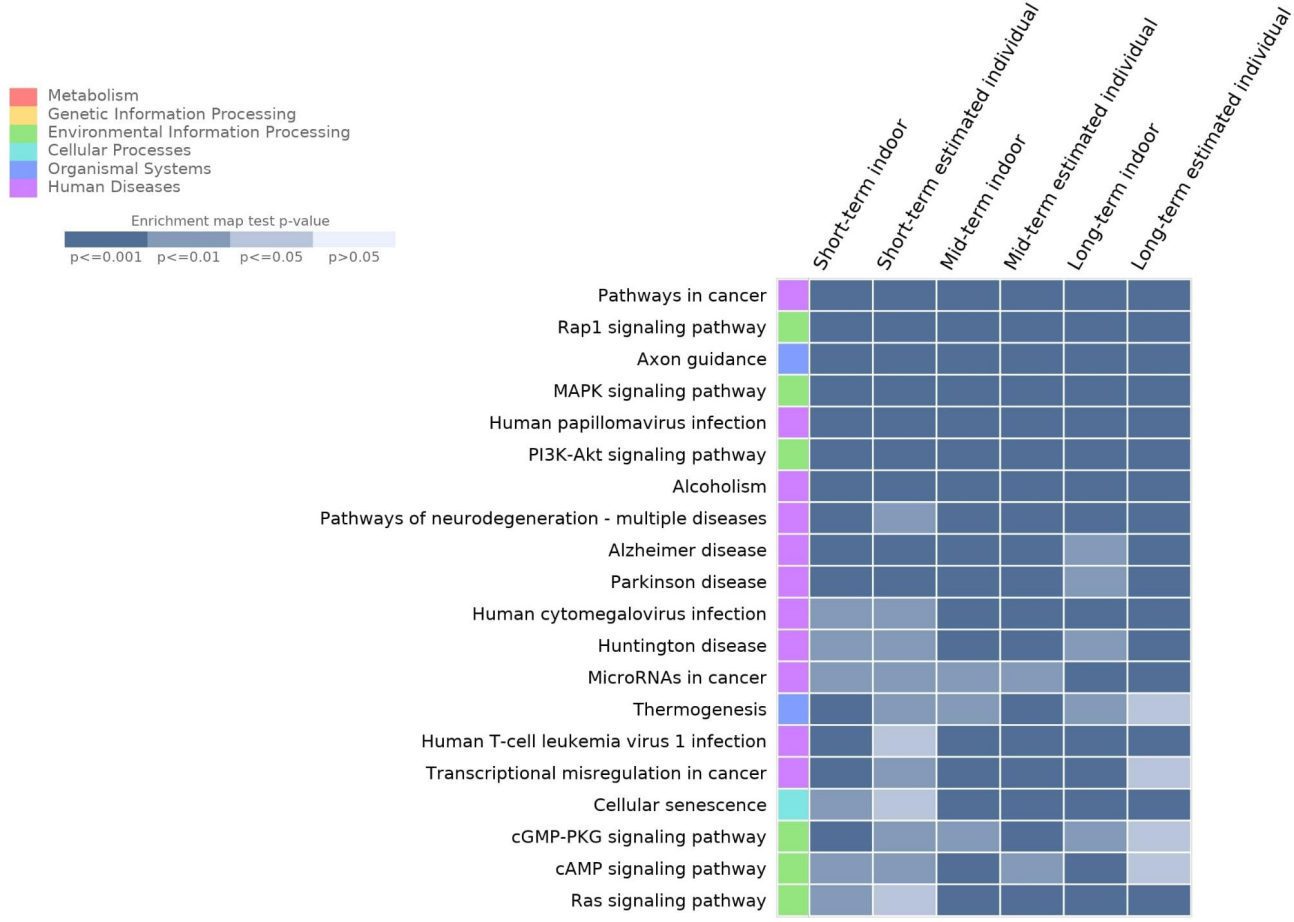
Results of Kyoto Encyclopedia of Genes and Genomes pathway enrichment analysis. Top 20 Kyoto Encyclopedia of Genes and Genomes pathways related to differentially methylated genes depending on particulate matter exposure

Additionally, we performed pathway analysis based on the KEGG database to identify pathways related to genes that were differentially methylated in response to PM_2.5_ exposure. Unlike previous GO-based functional enrichment analyses, several pathways related to neurodegenerative diseases, such as Alzheimer’s disease, Parkinson’s disease, and cancer, were identified, regardless of the PM_2.5_ exposure period (Fig. 4). The enrichment analysis identified a total of 68 KEGG pathways (Figure S3).

## Discussion

In this study, we used the methyl-capture method to explore the association between personal PM_2.5_ exposure and peripheral blood DNA methylation in patients with COPD. We found a positive association (hypermethylation) between DNA methylation in the promoter regions and PM_2.5_ exposure. Three different cumulative exposure windows were considered namely, 7, 35, and 90 days, representing short-, mid-, and long-term periods, respectively. Notably, the mid-term exposure window displayed the highest number of associations. Further, functional enrichment analysis revealed that the associated genes were mainly involved in neurodegenerative diseases and cancer pathways. This is the first study to investigate the association between PM_2.5_ exposure and DNA methylation in known CGIs in patients with indoor exposure throughout the study period and personal exposure based on portable measurement devices.

Studies have shown that averaging air pollution measured over longer time periods often results in stronger associations with DNA methylation changes [15]. Among the three different exposure periods, mid-term PM_2.5_ exposure was most frequently associated with differentially methylated CpGs in our study. Panni et al. investigated the effects of PM_2.5_ exposure on blood DNA methylation over different periods for up to 28 days and reported greater effects over a longer time window of exposure [16]. One study examined PM_2.5_ and NOx exposures, averaged over a full year, and their association with DNA methylation in circulating monocytes, revealing novel associations between long-term ambient air pollution exposure and site-specific DNA methylation [17]. The strength of our study is that it demonstrates the association and the relevant loci at three different periods.

Nevertheless, how PM translocation from the lungs to the blood induces DNA methylation is not yet fully understood. According to current knowledge, PM_2.5_ can induce oxidative stress and inflammation in cells, resulting in the production of reactive oxygen species and proinflammatory cytokines. These molecules can consequently affect the cellular machinery that regulates DNA methylation [11, 15]. Moreover, PM_2.5_ might directly interact with enzymes that add or remove methyl groups from DNA, such as DNA methyltransferases or translocation proteins. The altered activity of these enzymes can change DNA methylation pattern [18, 19]. The process of DNA methylation requires the presence of methyl groups derived from molecules such as S-adenosylmethionine. Exposure to PM_2.5_ may disrupt the metabolism of these molecules, thereby indirectly influencing DNA methylation [15].

We identified various hypermethylated genes associated with PM_2.5_ exposure, many of which are known to be related to human health. *TMTC2* has been identified as a candidate for causing progressive sensory hearing loss in humans [20, 21]. *GLRX3* is a major redox buffer that uses the reducing power of glutathione to maintain and regulate the cellular redox state [22]. It protects the lung tissue from oxidative stress, and an altered *GLRX3* is known to affect idiopathic pulmonary fibrosis, asthma, and COPD in rodent models or cell-based studies [23]. Moreover, levels of *GLRX3* are significantly increased in lung cancer tissues [22]. CUL4A comprises the multifunctional ubiquitin ligase E3 complex, where specific DDB1 and CUL4-associated factors (*DCAFs*) determine substrate specificity. *DCAFs* serve as substrate receptors that execute the degradation of proteins [24]. Alterations encompassing *DCAFs* are frequently observed in lung adenocarcinoma, and *DCAF15* has been shown to be frequently lost [25]. Additionally, we identified TMTC2, an integral membrane protein associated with the endoplasmic reticulum calcium uptake pump; however, complete details regarding its function are not yet known.

CCR4-NOT transcription complex subunit 6 like (*CNOT6L*) is a deadenylase subunit belonging to the CCR4-NOT complex, a major deadenylase complex in eukaryotes [26]. The function of *CNOT6L* has not been elucidated; however, one previous study demonstrated a significant copy number loss of *CNOT6L* in human colon adenocarcinoma samples [27]. The expression of *CNOT6L* was reportedly downregulated in samples of leukemia cells from patients with acute lymphoblastic and myeloid leukemia compared to that in normal blood cells [28]. Bassoon (BSN) is a presynaptic scaffolding protein involved in organizing the presynaptic cytoskeleton. This gene is primarily expressed in the neurons of the brain. Mutations in *BSN* have been reported in individuals with familial and sporadic progressive supranuclear palsy-like syndrome [29]. SENP6 is a ubiquitin-like molecule that serves as a key factor required throughout the cell cycle and controls centromere stability [30]. Genetic alterations or instability in *SENP6* have been reported in lymphomagenesis and diffuse large B-cell lymphoma [31]. All these data suggest that epigenetic change can be the underlying pathogenic mechanism of PM_2.5_ exposure-mediated effects.

Changes in DNA methylation may be associated with the development and exacerbation of lung diseases. In Boston, a 28-day average exposure to PM_2.5_ resulted in significantly decreased lung function, measured in terms of FEV_1_ and FVC. These associations were significantly stronger among participants with higher methylation at CpG sites on the glucocorticoid receptor. Moreover, associations of PM_2.5_ with FVC were significantly stronger among participants with lower methylation at one of the five CpG sites in Toll-like receptor 2 [32]. A large Dutch population-based cohort study identified differential DNA methylation at seven CpG sites with a genome-wide significant association with NO_2_ exposure. Although a genome-wide significant effect of PM_2.5_ exposure on DNA methylation related to lung function was not found, many CpG sites had suggestive effects in response to PM_2.5_ [33].

The enrichment analysis performed in this study demonstrated a strong association between pathways in cancer and progressive neurological diseases, such as Parkinson’s and Alzheimer’s diseases, in all three different cumulative exposure windows. Among the biological and cellular processes, the modulation and regulation of synapses were significantly associated with mid-term exposure. A relationship between PM_2.5_-derived hypomethylation and Alzheimer’s disease, especially methylation changes associated with amyloid precursor protein, beta-site amyloid precursor protein cleaving enzyme 1, and the apolipoprotein E gene, has also been reported [34, 35]. Moreover, clinical studies on prolonged exposure to PM_2.5_ have demonstrated that DNA hypomethylation and abnormal glutathione pathways lead to epigenetic changes and trigger neuroinflammation and clearance of reactive oxygen species [36]. Compared to Alzheimer’s disease, evidence about the association between PM_2.5_-derived methylation and Parkinson’s disease is limited [37].

Our study has some limitations. First, our results have not been validated in other populations. Second, specifically with respect to COPD, owing to the lack of a control group, we are not certain whether these results are exclusive findings or generalized in the older population. Moreover, the results cannot be generalized to women with COPD. Third, we did not measure the expression levels of the differentially methylated genes. An estimation of the protein or mRNA expression of these genes may help elucidate the functional and clinical impact of genetic methylation on PM_2.5_ exposure in the context of COPD. Fourth, if we had analyzed the various PM categories, it could have provided additional information about differential DNA methylation. However, among the various PM categories, PM_2.5_ is mostly deposited in small airways and it is closely related to clinical parameters in COPD [38, 39]. Moreover, CP-16-A5 (Aircok, Seoul, Republic of Korea) was most suitable IoT-based device for monitoring as closely and in detail as possible, which focused on measuring PM_2.5_. Lastly, we only considered effects of PM_2.5_ on DNA methylation among the air pollution components. However, air pollution is also composed of black carbon, ozone, nitrogen oxides, and polyaromatic hydrocarbons. These constituents are known to be associated with changes in DNA methylation leading to the lung function [15].

Despite these limitations, our study has several strengths. We measured personal PM_2.5_ exposure using an individualized portable device, whereas previous studies estimated PM_2.5_ exposure using fixed monitoring stations with low spatial resolution. Such measurements can have limitations if the number of monitoring stations is limited. Moreover, we analyzed the association between DNA methylation and indoor PM_2.5_ levels over a study period of 1 year to minimize seasonal variation. Additionally, older adults with chronic diseases usually perform fewer outdoor activities, and indoor sources might be larger contributors to personal exposure in them.

## Conclusions

We demonstrated the association between DNA methylation and PM_2.5_ exposure in three different cumulative exposure windows. Significant associations were observed even in short-term exposure, whereas the extent of DNA methylation was highest in mid-term exposure. Biologically, synaptic transmission in neurodegenerative diseases and various pathways in cancer were most affected in patients with COPD. Our study provides a better understanding of the effects of PM_2.5_ exposure linked to adverse health outcomes in patients with COPD. Replication of our findings in further studies is necessary to elucidate the role of suggested epigenetic changes associated with PM_2.5_ exposure.

## Electronic supplementary material

Below is the link to the electronic supplementary material.

Supplementary Material 1

Supplementary Material 2

## Data Availability

The datasets used and analyzed during the current study are available from the corresponding author on reasonable request.

## Abbreviations

PM_2.5_: Particulate matter with a diameter of < 2.5 μm
DALY: Disability-adjusted life years
COPD: Chronic obstructive pulmonary disease
CpG: 5′-C-phosphate-G-3′
FEV_1_: Forced expiratory volume in 1 s
FVC: Force vital capacity
