# Supplementary material for: The association between cumulative exposure to PM2.5 and DNA methylation measured using methyl-capture sequencing among COPD patients
Source: Respir Res. 2024 Sep 9;25:335. doi: 10.1186/s12931-024-02955-3 (PMC11386081; doi:10.1186/s12931-024-02955-3)

Supplementary Material

Methods

Estimation of individual $PM_{2.5}$ exposure levels

Land use regression model, a widely used technique for exposure estimation, was employed to estimate unmeasured $PM_{2.5}$ exposures. A microenvironmental model called Stochastic Human Exposure and Dose Simulation was applied to determine the distributions of microenvironmental $PM_{2.5}$ concentrations and exposures to patients. Individual $PM_{2.5}$ exposure levels were evaluated by calculating the 3-month time-weighted exposure using the following equation:

$$E = (C_{hin} \times T_{hin} + C_{hout} \times T_{hout} + C_{out} \times T_{out} + IF_{h} \times (C_{out} \times T_{in}) + T_{trav} \times IF_{t} \times (C_{hout} + C_{out})/2)/24$$

In the provided equation, the time scale is expressed in days. Indoor and outdoor $PM_{2.5}$ concentrations denoted as C_{hin} and C_{hout}, are measured using IoT-based devices. C_{out} represents the estimated outdoor $PM_{2.5}$ concentrations based on the LUR model for unmeasured $PM_{2.5}$ exposure. T_{hin} and T_{hout} represent the time spent inside and outside of the participants' homes while T_{in} and T_{out} represent the time spent inside and outside other places, respectively. T_{trav} signifies the time spent on travel or commuting. Infiltration factors for ambient air pollutants entering indoors and various traffic vehicles are represented by IF_{h} and IF_{t}, respectively. Exposure on different traffic vehicles was estimated by the average level of infiltrated ambient pollutants outside residences and other places [$(C_{hout} + C_{out})/2$].

DNA methylation profiling

Sampling and library construction

In this study, we performed DNA methylation analysis using the blood samples obtained at the last follow-up visit. The genomic DNA isolated from the sample was checked for integrity using anagarose gel electrophoresis and quantified using PicoGreen™ (Invitrogen). The fragmentation of genomic DNA was performed using a focused ultrasonicator (Covaris). The fragmented DNA was repaired, an 'A' was ligated to the 3′ end, and SureSelect Methyl-Seq Methylated Adapters were ligated to the fragments. Once ligation was assessed, the adapter-ligated product was amplified using PCR. The final purified product was quantified using qPCR, according to the qPCR Quantification Protocol Guide, and quantitatively analyzed using TapeStation DNA Screentape D1000 (Agilent). For target capture, 250 ng of DNA was mixed with hybridization buffers, blocking mixes, RNase block, and 5 µl of SureSelect All DNA methylation region Capture Library, according to the standard SureSelect Methyl-Seq Target Enrichment protocol (Agilent). Hybridization of the capture baits was conducted at 65 °C with the heated thermal cycler lid option at 105 °C for 24 h using a PCR machine. The SureSelect Human Methyl-Seq kit captured 84.4 Mb of the human genome. Hybrids were captured on streptavidin beads, and the captured genomic DNA was eluted. Unmethylated C residues were modified using bisulfite conversion with the EZ DNA Methylation Gold kit (Zymo Research). Sequence-modified target-enriched libraries were indexed for multiplexing. The final libraries were pooled, clustered on a paired-end read-flow cell, and sequenced on an Illumina NovaSeq 6000 System for 2×101 cycles, at a depth of approximately 100 M reads per sample.

Methylation calling and data preprocessing

Supplementary Figure 1 shows the analytical methods and workflow. After sequencing, the raw sequence reads were trimmed based on low base quality and adapter sequences using Trimmomatic (version 0.38).[1] Next, the trimmed reads were aligned to the *Homo sapiens* hg19 reference genome using BSMAP (version 2.90; parameter set -n 1 -r 0), allowing only uniquely mapped reads. The mapped reads (in SAM file format) were sorted and indexed, and PCR duplicates were removed using SAMBAMBA (version 0.6.5). The methylation ratio of every single cytosine location within an on-target region was then extracted from the mapping results using the "methylratio.py" script in BSMAP. The coverage profiles were calculated as C counts/effective CT counts for each cytosine in CpG, CHH, and CHG. Each cytosine locus in CpG, CHH, and CHG was annotated using National Center for Biotechnology Information RefSeq gene annotation (NCBI_105.20190906). Annotation included the functional location of each gene (promoter regions, defined as -2 kb upstream of the transcription start site, exons, and introns), transcript ID, gene ID, strand, and CpG islands.

For data preprocessing, we selected only CpG sites with at least 10 CT counts at each site to

obtain a more reliable methylation ratio. The methylation ratio data were normalized using the median scaling normalization method to reduce technical bias and render the data samples more comparable.

Model selection

To check all the assumptions of a linear regression model (linearity, independence, homoscedasticity, and normality), we randomly selected 500 CpG sites and checked the following: whether a linear relationship exists between the dependent and independent variables in a scatter plot, whether the residuals were independent, whether the variance of the residuals was constant across the values of the independent variable (using the studentized Breusch–Pagan test), and whether the residuals followed a normal distribution (using the Shapiro–Wilk normality test). As most of the selected CpGs did not satisfy the assumptions of homoscedasticity or normality, we considered mixed-effects models with fixed and random effects to predict the dependent variable across different independent variable values. To determine the optimal model for all CpG sites, we randomly selected 500 CpGs and performed the following iterative process:

1. Fitted the full model
2. Adjusted the fixed effect, random effect, and variance functions
3. The new mixed-effects model was compared with the old model using the lower Akaike information criterion or likelihood-ratio test
4. The non-significant terms were removed from the fixed effects
5. Back to step 2

In this process, the independent variables—particulate matter exposure, age (years), body-mass index (kg/m^2), asthma (diagnosed or not diagnosed), and FEV_1 predicted %—were chosen as the fixed effects. Repeated measure days (7, 14, 21, 35, and 90 days) or asthma status were chosen as the variance covariates, whereas the COVID_hx variable was selected as the random effect. We evaluated the model with a lower Akaike information criterion score as the better-fit model.

Reference

1 Bolger AM, Lohse M, Usadel B. Trimmomatic: a flexible trimmer for Illumina sequence data. *Bioinformatics* 2014;30:2114-20.

Figure S1. Analysis and workflow

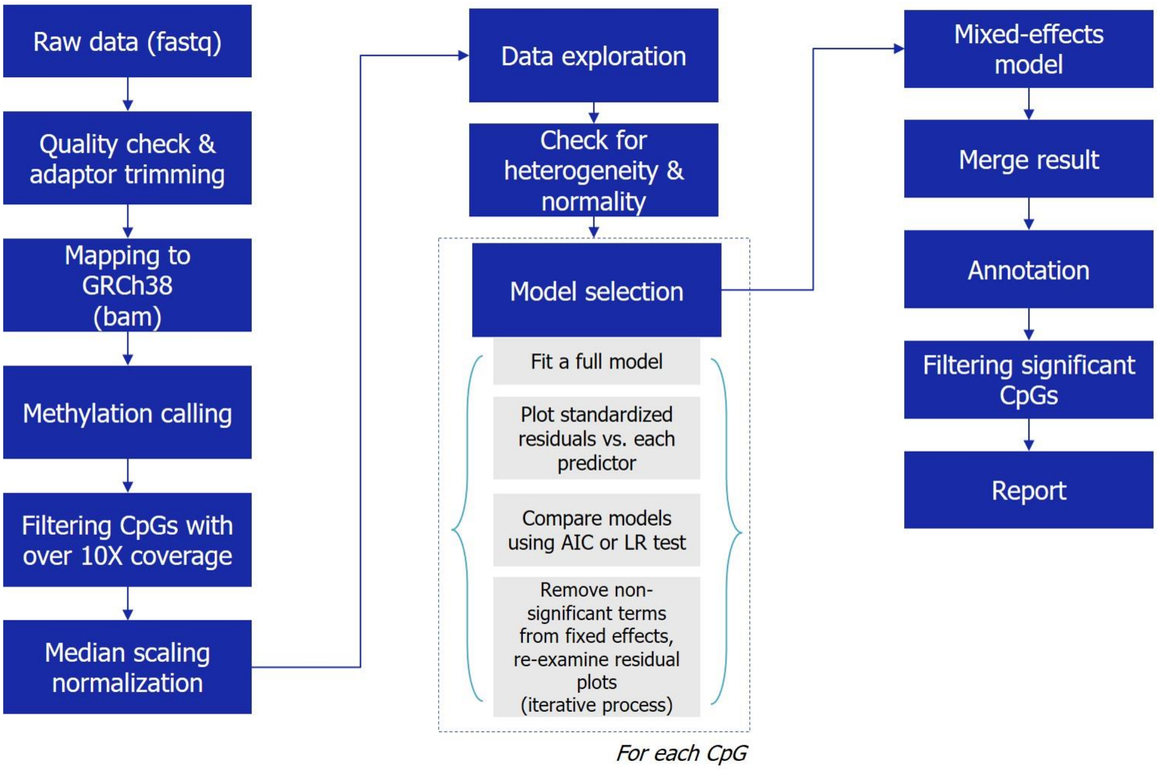

Table S1. Characteristics of differentially methylated CpG associated with $PM_{2.5}$ exposure in the genome-wide methylation analysis

Chromosome	Locus	Gene	Region	CGI type	adj. *p*-value	Marginal R^2	Conditional R^2
**Short-term exposure**							
5q13.2	chr05-72715694	*LINC02230, FOXD1*	Intergenic	CGI	7.72×10^{-3}	0.436	0.436
**Mid-term exposure**							
1q32.1	chr01-201969710	*RNPEP; ELF3-AS1*	Intronic; ncRNA_intronic	.	3.08×10^{-3}	0.412	0.412
1q42.12	chr01-226496435	*LIN9*	Intronic	CGI	3.74×10^{-4}	0.412	0.417
1p31.1	chr01-72302753	*NEGR1*	Intronic	.	6.32×10^{-3}	0.413	0.413
2q14.3	chr02-127904944	*BIN1, CYP27C1*	Intergenic	.	6.29×10^{-3}	0.407	0.407
2q32.2	chr02-190445635	*SLC40A1*	Upstream	CGI	9.18×10^{-4}	0.419	0.419
2p13.3	chr02-71295708	*NAGK*	Intronic	CGI	3.50×10^{-4}	0.496	0.496
3q23	chr03-142683000	*PAQR9-AS1*	ncRNA_intronic	CGI	1.37×10^{-3}	0.427	0.427
3p21.31	chr03-50192500	*SEMA3F*	UTR5	CGI	7.29×10^{-4}	0.454	0.454
4q12	chr04-57522507	*HOPX*	UTR5	CGI	2.63×10^{-3}	0.430	0.430
4q13.2	chr04-69215327	*YTHDC1*	Intronic	CGI	2.92×10^{-3}	0.411	0.411
5p15.31	chr05-6687397	*LINC02102*	ncRNA_intronic	.	1.06×10^{-3}	0.408	0.408
5q13.2	chr05-72571285	*LOC340090, LINC02230*	Intergenic	.	1.06×10^{-3}	0.459	0.459
6q21	chr06-106426022	*LOC100130683, PRDM1*	Intergenic	Shelf	1.59×10^{-4}	0.464	0.463
6p22.2	chr06-26285655	*H4C8*	Exonic	CGI	3.74×10^{-4}	0.426	0.426
7p22.3	chr07-1535839	*INTS1*	Exonic	Shelf	3.61×10^{-3}	0.402	0.402
8q21.3	chr08-87495233	*NTAN1P2*	ncRNA_exonic	.	1.46×10^{-4}	0.486	0.486
10q24.32	chr10-103990556	*PITX3*	Exonic	CGI	4.10×10^{-3}	0.401	0.401
10q24.2	chr10-99474170	*MARVELD1*	UTR3	Shore	5.43×10^{-3}	0.442	0.442
11q23.2	chr11-112833348	*NCAM1; LOC101928847*	Intronic; ncRNA_intronic	CGI	1.46×10^{-4}	0.413	0.413
11q12.2	chr11-61595226	*FADS2*	Intronic	CGI	3.62×10^{-3}	0.359	0.412
12q24.33	chr12-133020939	*LOC101928416, FBRSL1*	Intergenic	Shore	2.01×10^{-3}	0.406	0.406
13q33.3	chr13-107188237	*EFNB2*	Upstream	CGI	3.44×10^{-4}	0.481	0.481
13q34	chr13-113472663	*ATP11A*	Intronic	CGI	2.52×10^{-4}	0.484	0.484
14q23.3	chr14-64932286	*AKAP5*	UTR5	CGI	2.43×10^{-4}	0.441	0.441
16q12.1	chr16-50745234	*NOD2*	Exonic	.	1.45×10^{-3}	0.469	0.469
17p11.2	chr17-21356194	*KCNJ12, LINC02693*	Intergenic	CGI	1.37×10^{-3}	0.432	0.432
17p13.2	chr17-4643219	*ZMYND15; CXCL16*	UTR5	CGI	7.29×10^{-4}	0.462	0.462

22q13.31	chr22-45097844	*PRR5*	Intronic	CGI	3.44×10^{-4}	0.405	0.405
Xq22.2	chrX-102984081	*GLRA4*	Upstream	.	3.74×10^{-4}	0.446	0.446
Xq13.1	chrX-68049583	*EFNB1*	UTR5	CGI	1.31×10^{-3}	0.471	0.471
**Long-term exposure**							
17q11.2	chr17-29421733	*MIR4733*	Upstream	CGI	1.44×10^{-3}	0.415	0.415

Abbreviations: CpG, 5′-C-phosphate-G-3′; $PM_{2.5}$, particulate matter <2.5 micrometers in diameter; CGI, CpG island; UTR, untranslated region; ncRNA, non-coding ribonucleic acid

Figure S2. Gene-ontology-based functional enrichment analysis

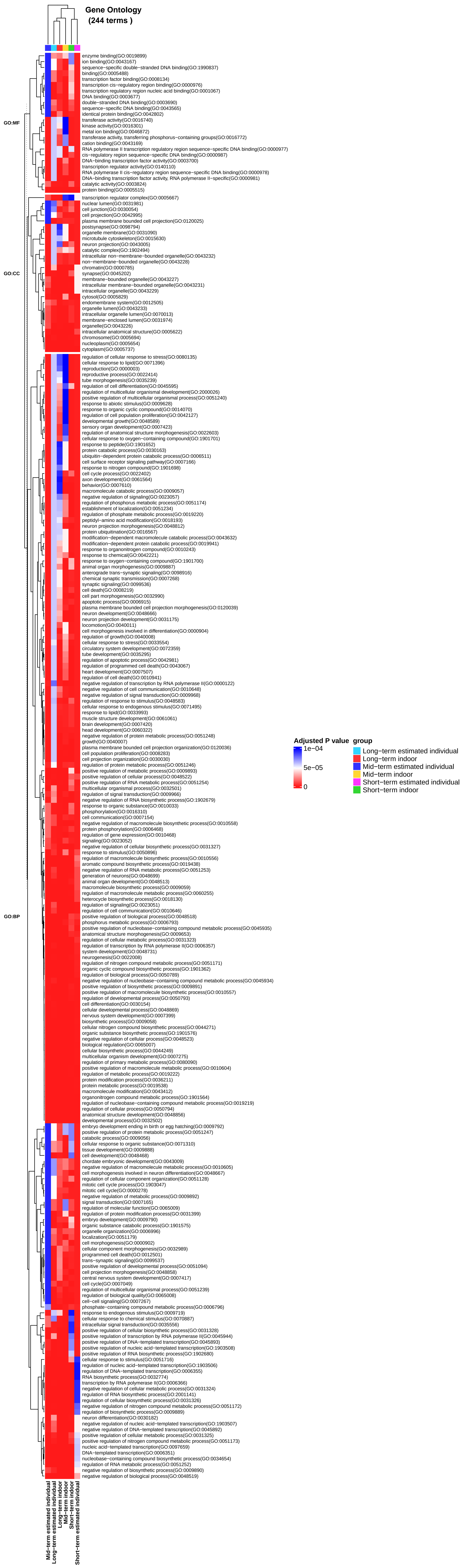

Figure S3. Pathway analysis based on the KEGG

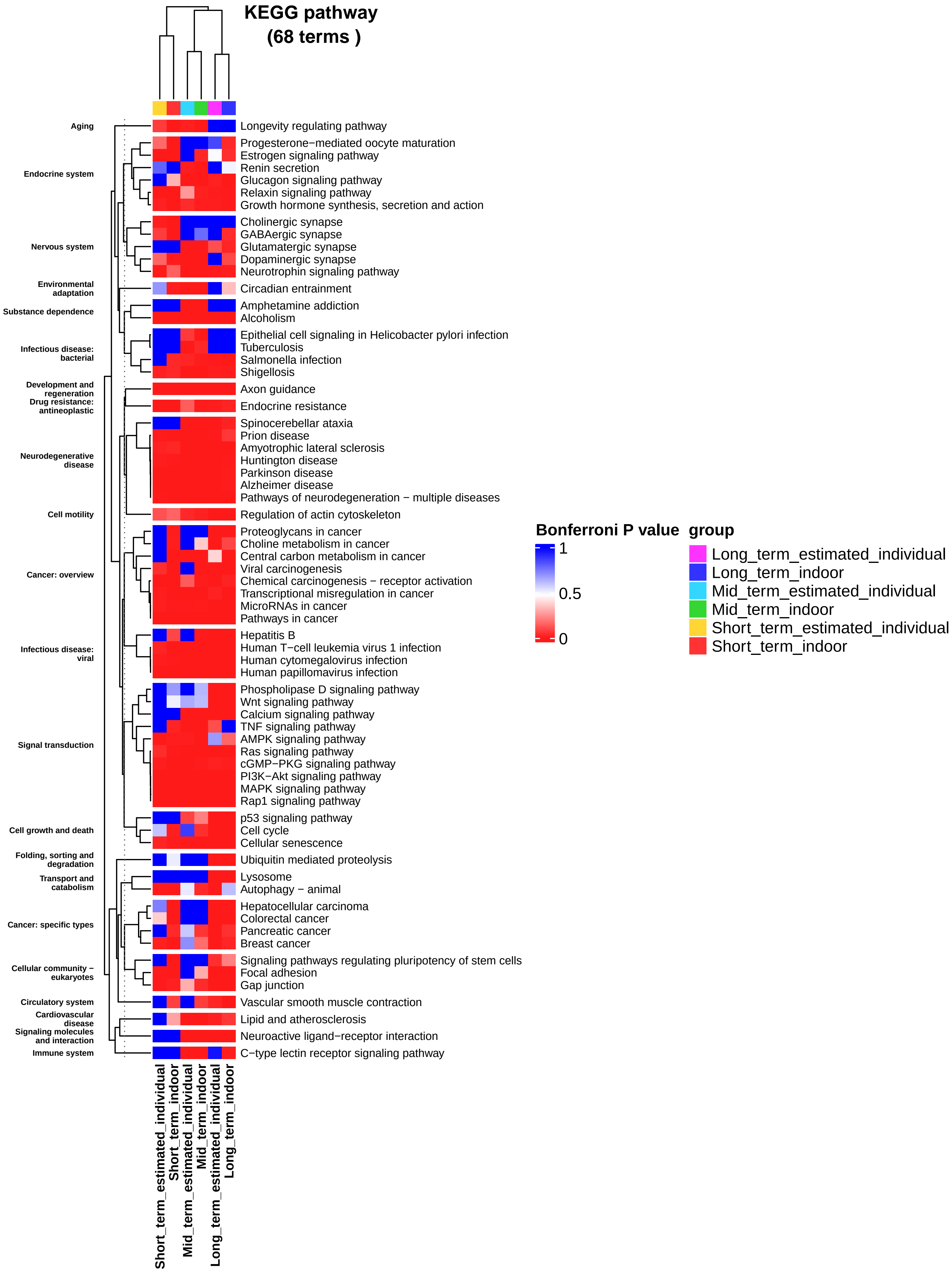

Supplement: Supplementary file 1 — Supplementary Material 1 [file 12931_2024_2955_MOESM1_ESM.pdf]
