# Supplementary material for: The association between cumulative exposure to PM2.5 and DNA methylation measured using methyl-capture sequencing among COPD patients
Source: Respir Res. 2024 Sep 9;25:335. doi: 10.1186/s12931-024-02955-3 (PMC11386081; doi:10.1186/s12931-024-02955-3)
Supplement: Supplementary file 2 — Supplementary Material 2 [file 12931_2024_2955_MOESM2_ESM.docx]

| Table S1. Characteristics of differentially methylated CpG associated with PM_2.5_ exposure in the genome-wide methylation analysis | | | | | | | |
| --- | --- | --- | --- | --- | --- | --- | --- |
| Chromosome | **Locus** | **Gene** | **Region** | **CGI type** | **adj. *p*-value** | **Marginal R^2^** | **Conditional R^2^** |
| Short-term exposure | | | | | | | |
| 5q13.2 | chr05-72715694 | *LINC02230, FOXD1* | Intergenic | CGI | 7.72 x 10^-3^ | 0.436 | 0.436 |
|  |  |  |  |  |  |  |  |
| Mid-term exposure | | | | | | | |
| 1q32.1 | chr01-201969710 | *RNPEP; ELF3-AS1* | Intronic; ncRNA_intronic | . | 3.08 x 10^-3^ | 0.412 | 0.412 |
| 1q42.12 | chr01-226496435 | *LIN9* | Intronic | CGI | 3.74 x 10^-4^ | 0.412 | 0.417 |
| 1p31.1 | chr01-72302753 | *NEGR1* | Intronic | . | 6.32 x 10^-3^ | 0.413 | 0.413 |
| 2q14.3 | chr02-127904944 | *BIN1, CYP27C1* | Intergenic | . | 6.29 x 10^-3^ | 0.407 | 0.407 |
| 2q32.2 | chr02-190445635 | *SLC40A1* | Upstream | CGI | 9.18 x 10^-4^ | 0.419 | 0.419 |
| 2p13.3 | chr02-71295708 | *NAGK* | Intronic | CGI | 3.50 x 10^-4^ | 0.496 | 0.496 |
| 3q23 | chr03-142683000 | *PAQR9-AS1* | ncRNA_intronic | CGI | 1.37 x 10^-3^ | 0.427 | 0.427 |
| 3p21.31 | chr03-50192500 | *SEMA3F* | UTR5 | CGI | 7.29 x 10^-4^ | 0.454 | 0.454 |
| 4q12 | chr04-57522507 | *HOPX* | UTR5 | CGI | 2.63 x 10^-3^ | 0.430 | 0.430 |
| 4q13.2 | chr04-69215327 | *YTHDC1* | Intronic | CGI | 2.92 x 10^-3^ | 0.411 | 0.411 |
| 5p15.31 | chr05-6687397 | *LINC02102* | ncRNA_intronic | . | 1.06 x 10^-3^ | 0.408 | 0.408 |
| 5q13.2 | chr05-72571285 | *LOC340090, LINC02230* | Intergenic | . | 1.06 x 10^-3^ | 0.459 | 0.459 |
| 6q21 | chr06-106426022 | *LOC100130683, PRDM1* | Intergenic | Shelf | 1.59 x 10^-4^ | 0.464 | 0.463 |
| 6p22.2 | chr06-26285655 | *H4C8* | Exonic | CGI | 3.74 x 10^-4^ | 0.426 | 0.426 |
| 7p22.3 | chr07-1535839 | *INTS1* | Exonic | Shelf | 3.61 x 10^-3^ | 0.402 | 0.402 |
| 8q21.3 | chr08-87495233 | *NTAN1P2* | ncRNA_exonic | . | 1.46 x 10^-4^ | 0.486 | 0.486 |
| 10q24.32 | chr10-103990556 | *PITX3* | Exonic | CGI | 4.10 x 10^-3^ | 0.401 | 0.401 |
| 10q24.2 | chr10-99474170 | *MARVELD1* | UTR3 | Shore | 5.43 x 10^-3^ | 0.442 | 0.442 |
| 11q23.2 | chr11-112833348 | *NCAM1; LOC101928847* | Intronic; ncRNA_intronic | CGI | 1.46 x 10^-4^ | 0.413 | 0.413 |
| 11q12.2 | chr11-61595226 | *FADS2* | Intronic | CGI | 3.62 x 10^-3^ | 0.359 | 0.412 |
| 12q24.33 | chr12-133020939 | *LOC101928416, FBRSL1* | Intergenic | Shore | 2.01 x 10^-3^ | 0.406 | 0.406 |
| 13q33.3 | chr13-107188237 | *EFNB2* | Upstream | CGI | 3.44 x 10^-4^ | 0.481 | 0.481 |
| 13q34 | chr13-113472663 | *ATP11A* | Intronic | CGI | 2.52 x 10^-4^ | 0.484 | 0.484 |
| 14q23.3 | chr14-64932286 | *AKAP5* | UTR5 | CGI | 2.43 x 10^-4^ | 0.441 | 0.441 |
| 16q12.1 | chr16-50745234 | *NOD2* | Exonic | . | 1.45 x 10^-3^ | 0.469 | 0.469 |
| 17p11.2 | chr17-21356194 | *KCNJ12, LINC02693* | Intergenic | CGI | 1.37 x 10^-3^ | 0.432 | 0.432 |
| 17p13.2 | chr17-4643219 | *ZMYND15; CXCL16* | UTR5 | CGI | 7.29 x 10^-4^ | 0.462 | 0.462 |
| 22q13.31 | chr22-45097844 | *PRR5* | Intronic | CGI | 3.44 x 10^-4^ | 0.405 | 0.405 |
| Xq22.2 | chrX-102984081 | *GLRA4* | Upstream | . | 3.74 x 10^-4^ | 0.446 | 0.446 |
| Xq13.1 | chrX-68049583 | *EFNB1* | UTR5 | CGI | 1.31 x 10^-3^ | 0.471 | 0.471 |
|  | | | | | | | |
| Long-term exposure | | | | | | | |
| 17q11.2 | chr17-29421733 | *MIR4733* | Upstream | CGI | 1.44 x 10^-3^ | 0.415 | 0.415 |
| Abbreviations: CpG, 5′-C-phosphate-G-3′; PM_2.5_, particulate matter <2.5 micrometers in diameter; CGI, CpG island; UTR, untranslated region; ncRNA, non-coding ribonucleic acid | | | | | | | |
